# Antibody Response After the Third SARS-CoV-2 Vaccine in Solid Organ Transplant Recipients and People Living With HIV (COVERALL-2)

**DOI:** 10.1093/ofid/ofad536

**Published:** 2023-11-03

**Authors:** Alexandra Griessbach, Frédérique Chammartin, Irene A Abela, Patrizia Amico, Marcel P Stoeckle, Anna L Eichenberger, Barbara Hasse, Dominique L Braun, Macé M Schuurmans, Thomas F Müller, Michael Tamm, Annette Audigé, Nicolas J Mueller, Andri Rauch, Huldrych F Günthard, Michael T Koller, Alexandra Trkola, Selina Epp, Alain Amstutz, Christof M Schönenberger, Ala Taji Heravi, Matthaios Papadimitriou-Olivgeris, Alessio Casutt, Oriol Manuel, Katharina Kusejko, Heiner C Bucher, Matthias Briel, Benjamin Speich, the Swiss HIV Cohort Study and the Swiss Transplant Cohort Study, Abela I, Aebi-Popp K, Anagnostopoulos A, Battegay M, Bernasconi E, Braun DL, Bucher HC, Calmy A, Cavassini M, Ciuffi A, Dollenmaier G, Egger M, Elzi L, Fehr J, Fellay J, Furrer H, Fux CA, Günthard HF, Hachfeld A, Haerry D, Hasse B, Hirsch HH, Hoffmann M, Hösli I, Huber M, Jackson-Perry D, Kahlert CR, Kaiser L, Keiser O, Klimkait T, Kouyos RD, Kovari H, Kusejko K, Labhardt N, Leuzinger K, Martinez de Tejada B, Marzolini C, Metzner KJ, Müller N, Nemeth J, Nicca D, Notter J, Paioni P, Pantaleo G, Perreau M, Rauch A, Salazar-Vizcaya L, Schmid P, Speck R, Stöckle M, Tarr P, Trkola A, Wandeler G, Weisser M, Yerly S, Patrizia Amico, John-David Aubert, Vanessa Banz, Sonja Beckmann, Guido Beldi, Christoph Berger, Ekaterine Berishvili, Annalisa Berzigotti, Isabelle Binet, Pierre-Yves Bochud, Sanda Branca, Heiner Bucher, Emmanuelle Catana, Anne Cairoli, Yves Chalandon, Sabina De Geest, Olivier De Rougemont, Sophie De Seigneux, Michael Dickenmann, Joëlle Lynn Dreifuss, Michel Duchosal, Thomas Fehr, Sylvie Ferrari-Lacraz, Christian Garzoni, Déla Golshayan, Nicolas Goossens, Fadi Haidar, Jörg Halter, Dominik Heim, Christoph Hess, Sven Hillinger, Hans H Hirsch, Patricia Hirt, Linard Hoessly, Günther Hofbauer, Uyen Huynh-Do, Franz Immer, Michael Koller, Bettina Laesser, Frédéric Lamoth, Roger Lehmann, Alexander Leichtle, Oriol Manuel, Hans-Peter Marti, Michele Martinelli, Valérie McLin, Katell Mellac, Aurélia Merçay, Karin Mettler, Nicolas J Mueller, Ulrike Müller-Arndt, Beat Müllhaupt, Mirjam Nägeli, Graziano Oldani, Manuel Pascual, Jakob Passweg, Rosemarie Pazeller, Klara Posfay-Barbe, Juliane Rick, Anne Rosselet, Simona Rossi, Silvia Rothlin, Frank Ruschitzka, Thomas Schachtner, Stefan Schaub, Alexandra Scherrer, Aurelia Schnyder, Macé Schuurmans, Simon Schwab, Thierry Sengstag, Federico Simonetta, Susanne Stampf, Jürg Steiger, Guido Stirnimann, Ueli Stürzinger, Christian Van Delden, Jean-Pierre Venetz, Jean Villard, Julien Vionnet, Madeleine Wick, Markus Wilhelm, Patrick Yerly

**Affiliations:** Division of Clinical Epidemiology, Department of Clinical Research, University Hospital Basel, University of Basel, Basel, Switzerland; Division of Clinical Epidemiology, Department of Clinical Research, University Hospital Basel, University of Basel, Basel, Switzerland; Institute of Medical Virology, University of Zurich, Zurich, Switzerland; Department of Infectious Diseases and Hospital Epidemiology, University Hospital Zurich, Zurich, Switzerland; Clinic for Transplantation Immunology and Nephrology, University Hospital Basel, Basel, Switzerland; Division of Infectious Diseases and Hospital Epidemiology, University Hospital Basel, University of Basel, Basel, Switzerland; Department of Infectious Diseases, Inselspital, Bern University Hospital, University of Bern, Bern, Switzerland; Department of Infectious Diseases and Hospital Epidemiology, University Hospital Zurich, Zurich, Switzerland; Institute of Medical Virology, University of Zurich, Zurich, Switzerland; Department of Infectious Diseases and Hospital Epidemiology, University Hospital Zurich, Zurich, Switzerland; Division of Pulmonology, University Hospital Zurich, Zurich, Switzerland; Nephrology Clinic, University Hospital Zurich, Zurich, Switzerland; Clinic of Respiratory Medicine and Pulmonary Cell Research, University Hospital Basel, Basel, Switzerland; Institute of Medical Virology, University of Zurich, Zurich, Switzerland; Clinic for Transplantation Immunology and Nephrology, University Hospital Basel, Basel, Switzerland; Department of Infectious Diseases, Inselspital, Bern University Hospital, University of Bern, Bern, Switzerland; Institute of Medical Virology, University of Zurich, Zurich, Switzerland; Department of Infectious Diseases and Hospital Epidemiology, University Hospital Zurich, Zurich, Switzerland; Clinic for Transplantation Immunology and Nephrology, University Hospital Basel, Basel, Switzerland; Swiss Transplant Cohort Study, University Hospital Basel, Basel, Switzerland; Institute of Medical Virology, University of Zurich, Zurich, Switzerland; Institute of Medical Virology, University of Zurich, Zurich, Switzerland; Division of Clinical Epidemiology, Department of Clinical Research, University Hospital Basel, University of Basel, Basel, Switzerland; Division of Clinical Epidemiology, Department of Clinical Research, University Hospital Basel, University of Basel, Basel, Switzerland; Division of Clinical Epidemiology, Department of Clinical Research, University Hospital Basel, University of Basel, Basel, Switzerland; Infectious Diseases Service and Transplantation Center, Lausanne University Hospital, Lausanne, Switzerland; Division of Pulmonology, Department of Medicine, University Hospital of Lausanne, Lausanne, Switzerland; Infectious Diseases Service and Transplantation Center, Lausanne University Hospital, Lausanne, Switzerland; Institute of Medical Virology, University of Zurich, Zurich, Switzerland; Department of Infectious Diseases and Hospital Epidemiology, University Hospital Zurich, Zurich, Switzerland; Division of Clinical Epidemiology, Department of Clinical Research, University Hospital Basel, University of Basel, Basel, Switzerland; Division of Infectious Diseases and Hospital Epidemiology, University Hospital Basel, University of Basel, Basel, Switzerland; Division of Clinical Epidemiology, Department of Clinical Research, University Hospital Basel, University of Basel, Basel, Switzerland; Department of Health Research Methods, Evidence, and Impact, McMaster University, Hamilton, Ontario, Canada; Division of Clinical Epidemiology, Department of Clinical Research, University Hospital Basel, University of Basel, Basel, Switzerland

**Keywords:** HIV, SARS-CoV-2, organ transplant, vaccine

## Abstract

**Background:**

After basic immunization with 2 mRNA SARS-CoV-2 vaccine doses, only a small proportion of patients who are severely immunocompromised generate a sufficient antibody response. Hence, we assessed the additional benefit of a third SARS-CoV-2 vaccine in patients with different levels of immunosuppression.

**Methods:**

In this observational extension of the COVERALL trial (Corona Vaccine Trial Platform), we recruited patients from the Swiss HIV Cohort Study and the Swiss Transplant Cohort Study (ie, lung and kidney transplant recipients). We collected blood samples before and 8 weeks after the third SARS-CoV-2 vaccination with either mRNA-1273 (Moderna) or BNT162b2 (Pfizer-BioNTech). The primary outcome was the proportion of participants showing an antibody response (Elecsys Anti-SARS-CoV-2 S test; threshold ≥100 U/mL) 8 weeks after the third SARS-CoV-2 vaccination. We also compared the proportion of patients who reached the primary outcome from basic immunization (the first and second vaccines) to the third vaccination.

**Results:**

Nearly all participants (97.2% [95% CI, 95.9%–98.6%], 564/580) had an antibody response. This response was comparable between mRNA-1273 (96.1% [95% CI, 93.7%–98.6%], 245/255) and BNT162b2 (98.2% [95% CI, 96.7%–99.6%], 319/325). Stratification by cohort showed that 99.8% (502/503) of people living with HIV and 80.5% (62/77) of recipients of solid organ transplants achieved the primary endpoint. The proportion of patients with an antibody response in solid organ transplant recipients improved from the second vaccination (22.7%, 15/66) to the third (80.5%, 62/77).

**Conclusions:**

People living with HIV had a high antibody response. The third vaccine increased the proportion of solid organ transplant recipients with an antibody response.

**Clinical Trials Registration.** NCT04805125 (ClinicalTrials.gov).

SARS-CoV-2 emerged late in 2019 in Wuhan, China, and induced a pandemic [1–3]. Approximately 1 year later, SARS-CoV-2 vaccines became available and were tested in large randomized placebo-controlled trials and determined safe and effective in terms of preventing COVID-19 [4, 5]. However, while the efficacy of vaccines was tested thoroughly in the general population, there was little evidence on vaccine protection in more vulnerable groups, such as patients who are immunocompromised [6]. Consequently, the Corona Vaccine Trial Platform (COVERALL) was established [7, 8]. COVERALL is a platform trial nested into the Swiss HIV Cohort Study (SHCS) [9] and the Swiss Transplant Cohort Study (STCS) [7, 10].

In the scope of the first COVERALL substudy (COVERALL-1) [11, 12], we randomized patients to the 2 available mRNA SARS-CoV-2 vaccines in Switzerland [13]: Pfizer-BioNTech (BNT162b2, Comirnaty) or Moderna (mRNA-1273, Spikevax). The study revealed that the vaccine response of Moderna was noninferior to Pfizer-BioNTech in terms of antibody response for patients who were immunocompromised [11]. While nearly all people living with HIV (PLWH) had an antibody response after 2 doses (Elecsys Anti-SARS-CoV-2 S test; threshold ≥100 U/mL), this was the case for only 24% of solid organ transplant (SOT) recipients. Previous evidence suggests that SOT recipients may benefit from a third vaccine [6, 14]. In late 2021, a third SARS-CoV-2 vaccine dose was recommended by Swiss health authorities to improve the protection of patients, as well as to account for genetic drift and emergence of new variants [13].

Hence, we initiated a new substudy to the COVERALL platform (COVERALL-2) in which we aimed to assess the benefit and potential harm of a third SARS-CoV-2 vaccine for patients who were immunocompromised among those recruited from the SHCS and the STCS. This observational study allowed for the inclusion of additional patients beyond the original randomized trial population (COVERALL-1).

## METHODS

### Study Oversight and Participants

The COVERALL platform study consists of 1 master protocol and 2 subprotocols. All protocols were approved by the ethical committee Nordwest- and Zentralschweiz, Switzerland (BASEC 2021-000593), and the full protocols are publicly available on trial registry (https://clinicaltrials.gov/ct2/show/NCT04805125).

This observational study (COVERALL-2) investigated the immune response after a third dose of SARS-CoV-2 vaccine among patients who were immunocompromised. Patients were enrolled in the following centers: University Hospital Basel (SHCS + STCS), University Hospital Zurich (SHCS + STCS), University Hospital Bern (SHCS), and University Hospital Lausanne (STCS). Patients were eligible to participate in the COVERALL-2 study if they were enrolled in 1 of the 2 cohorts (SHCS or STCS) and received the third SARS-CoV-2 vaccine dose within the frame of their clinical routine (see detailed inclusion and exclusion criteria in Supplementary Material 1 ).

### Vaccination and Data Collection

The third vaccine dose—BNT162b2 licensed by Pfizer-BioNTech (Comirnaty; 30 μg of BNT162b2 in 0.3 mL) or mRNA-1273 licensed by Moderna (Spikevax; 50 μg [SHCS] or 100 μg [STCS] of mRNA-1273 in 0.5 mL)—was given to the participants in the frame of their clinical routine, following the vaccine rollout program in Switzerland [13]. The study team collected blood samples (EDTA, 2 × 7.5 mL) at baseline (ie, up to 2 weeks before the third vaccination) and at the follow-up visit (ie, 8 weeks after the third vaccination; ±2 weeks). At the time of ethical approval, several patients had already been vaccinated, especially patients with a high risk for severe COVID-19 from the STCS. Therefore, we allowed study participation even if the baseline assessment was missing (ie, no baseline blood sample was available). Baseline variables (ie, before the third vaccination)—such as age, sex, history of cardiovascular or metabolic disease, CD4 T-cell counts, HIV viral load, immunosuppressive therapy, and time from transplant—were routinely collected from the corresponding cohort studies (SHCS and STCS). A test reactive to the nucleocapsid protein (Elecsys Anti-SARS-CoV-2; Roche Diagnostics) was also conducted at the baseline visit, indicating previous contact to SARS-CoV-2. Clinical outcomes and adverse events were assessed during the follow-up visit at 8 weeks (±2 weeks).

### Outcomes

The primary outcome was the proportion of patients with a positive antibody (pan-Ig) response to SARS-CoV-2 spike (S1) protein receptor-binding domain (RBD) in human serum or plasma, as assessed by the commercial immunoassay Elecsys Anti-SARS-CoV-2 S (Elecsys S) from Roche Diagnostics [15]. We used a cutoff ≥100 U/mL for antibody response, as indicated by Khoury et al [16]. Further immunologic endpoints were as follows:

- A sensitivity analysis with a threshold ≥0.8 U/mL for the Elecsys S test as defined by the manufacturer [17]
- An antibody response with the ABCORA 2 (Antibody Coronavirus Assay 2), which determines seropositivity by measuring IgG, IgA, and IgM responses to SARS-CoV-2 RBD in the S1 subunit of the spike protein, S1, S2, and N [15]
- Neutralization activity against the vaccine strain Wuhan-Hu-1 in sera, defined as having an ABCORA sum S1 (sum of S1 signal over cutoff values of IgG, IgA, IgM) above the threshold of 17 [15]
- Mean IgG response against RBD (pan-Ig anti–S1-RBD) of SARS-CoV-2
- Mean IgG, IgA and IgM to the SARS-CoV-2 S1 with ABCORA 2 (see Supplementary Material 2 for details)

Clinical outcomes consisted of the following:

- New polymerase chain reaction (PCR)– or antigen test–confirmed SARS-CoV-2 infections
- New PCR- or antigen test–confirmed symptomatic COVID-19
- New PCR- or antigen test–confirmed asymptomatic SARS-CoV-2 infection
- Severe COVID-19 defined as hospitalization or death due to COVID-19
- Patient-reported SARS-CoV-2 infections of household members

Due to a shift in COVID-19 testing practices in Switzerland, the first 3 prespecified clinical outcomes (ie, new PCR-confirmed SARS-CoV-2 infections) were adapted to include antigen test confirmed SARS-CoV-2 infections.

Safety outcomes included

- Any local symptom (redness, swelling, or prolonged pain at the injection site) limiting continuation of normal daily activities during the first 7 days after vaccination
- Any systemic symptoms (eg, fever, generalized muscle or joint pain) limiting continuation of normal daily activities during the first 7 days after vaccination
- Any vaccine-related symptoms leading to contacting a physician during the first 7 days after vaccination

Data management and collection were done with the REDCap electronic data capture tool [18].

### Sample Size

No formal sample size estimation was calculated for the present observational study. We invited the 430 participants of the original COVERALL study (COVERALL-1) to participate [11] and aimed to recruit additional ones from SHCS and STCS to increase our sample size and the precision of our estimates for this study extension (COVERALL-2).

### Analysis

Analyses were conducted on 2 data sets. In the first (“strict time window”), we included only those patients from whom we collected results within the prespecified time window (ie, 8 weeks after the third SARS-CoV-2 vaccination, allowing for a time window of ±2 weeks). In the second (“full data set”), we included all patients. We report the frequency, percentage, and Wald 95% confidence intervals (CIs) of serologic immune response to the third vaccine dose. We compared the responses between the vaccine groups using mean difference and 95% CI. No statistical tests were conducted. In addition, all outcomes were stratified by cohort study (SHCS or STCS). Immunologic outcomes were stratified by confirmed SARS-CoV-2 infection or not after the third vaccination. The primary outcome was analyzed by subgroups of interest. These included PLWH with a CD4 T-cell count ≥350 and <350 cells/μL and stratification of individuals by suppressed and unsuppressed HIV viral load (>50 copies/mL). For SOT recipients, we stratified by intense immunosuppressive therapy (triple or quadruple regimen) and less intense (dual regimen). Furthermore, we grouped all study participants according to sex (male or female), age (<60, 60–70, >70 years), and history of cardiovascular diseases or metabolic syndrome (see definition in Supplementary material).

Moreover, we assessed the mean immune response separately for PLWH with a CD4 T-cell count <350 cells/μL, PLWH with a CD4 T-cell count ≥350 cells/μL, lung transplant recipients, and kidney transplant recipients. For nonresponders (ie, patients without an antibody response ≥100 U/mL), we exploratorily assessed baseline characteristics, immunosuppression, and vaccine product. We also determined the number of participants who switched the vaccine product (eg, first 2 vaccines Pfizer-BioNTech, third vaccine Moderna). Finally, we compared the antibody response after the third SARS-CoV-2 vaccine with that after basic immunization (after the second vaccine), which was assessed in the previous randomized study (COVERALL-1) in the same patient population (SHCS and STCS) [11, 12].

Clinical outcomes such as COVID-19 confirmed by PCR or antigen test and patient-reported COVID-19 of household members were reported as frequency with percentage for the different vaccine products. The results solely based on the randomized sample of patients and their antibody responses after the third SARS-CoV-2 vaccine are presented in a separate short report [19]. While this report had data on 303 patients (277 SHCS and 26 STCS) who participated in COVERALL-1, the current study was enriched with additional patients from the 2 cohorts.

## RESULTS

Between 7 December 2021 and 21 March 2022, 601 participants were recruited in our observational study and received a third dose of the Moderna mRNA-1273 vaccine (44.4%, n = 267) or Pfizer-BioNTech BNT162b2 vaccine (55.6%, n = 334; Supplementary Figure 1).

Participants were recruited from the SHCS (85.9%, 516/601) and STCS (14.1%, 85/601). The majority of participants were male (75.9%, 456/601), and the median age was 56 years (IQR, 46–63; Table 1). At baseline, 14.3% (68/477, n = 124 missing baseline blood sample) of patients had a reactive antibody test result to the nucleocapsid protein, suggesting a previous SARS-CoV-2 infection. The majority of PLWH had CD4 cell counts >350 (92.2%; 476/516) and suppressed HIV viral load (96.9%, 500/516). Of the SOT recipients, approximately half were kidney transplant recipients (52.9%, 45/85) and the other half were lung transplant recipients (47.1%, 40/85). The majority of SOT recipients received intensive immunosuppressive therapy (87.1%, 74/85), whereas only 12.9% (11/85) were on a less intense regimen. Baseline data stratified by cohort study are presented in Supplementary Table 1.

**Table 1.** Baseline Characteristics Before Third Vaccination

	Median (IQR) or No. (%)		
Characteristic	Moderna	Pfizer-BioNTech	Total
Age, y	54 (46–61)	57 (46–64)	56 (46–63)
Sex			
Male	190/267 (71.2)	266/334 (79.6)	456/601 (75.9)
Female	77/267 (28.8)	68/334 (20.4)	145/601 (24.1)
Cohort			
SHCS	219/267 (82.0)	297/334 (88.9)	516/601 (85.9)
STCS	48/267 (18.0)	37/334 (11.1)	85/601 (14.1)
History of cardiovascular disease or metabolic syndrome			
No	165/267 (61.8)	207/334 (62.0)	372/601 (61.9)
Yes	102/267 (38.2)	127/334 (38.0)	229/601 (38.1)
CD4 cell count, cells/µL ^a^			
<350	15/219 (6.9)	25/297 (8.4)	40/516 (7.7)
≥350	204/219 (93.1)	272/297 (91.6)	476/516 (92.3)
Suppressed HIV viral load ^a,b^			
No	7/219 (3.2)	9/297 (3.0)	16/516 (3.1)
Yes	212/219 (96.8)	288/297 (97.0)	500/516 (96.9)
Transplanted organ ^c^			
Kidney	24/48 (50.0)	21/37 (56.8)	45/85 (52.9)
Lung	24/48 (50.0)	16/37 (43.2)	40/85 (47.1)
Immunosuppressive therapy ^c,d^			
Less intense (≤2 regimen)	6/48 (12.5)	5/37 (13.5)	11/85 (12.9)
Intense (3 or 4 regimen)	42/48 (87.5)	32/37 (86.5)	74/85 (87.1)
Time since transplant, d	1364 (396–3263)	1835 (355–2586)	1504 (355–2845)
Antibody test to the nucleocapside protein ^e^			
Nonreactive	168/192 (87.5)	241/285 (84.6)	409/477 (85.7)
Reactive	24/192 (12.5)	44/285 (15.4)	68/477 (14.3)
Missing	75/267 (28.1)	49/334 (14.7)	124/601 (20.6)

Abbreviations: SHCS, Swiss HIV Cohort Study; STCT, Swiss Transplant Cohort Study.

^a^Only patients from the SHCS.

^b^Unsuppressed HIV viral load defined as >50 copies/mL.

^c^Only patients from the STCT.

^d^Intense, triple or quadruple immunosuppressive regimen; less intense, dual immunosuppressive regimen.

^e^Elecsys N test reactive to nucleocapsid protein indicates previous contact to SARS-CoV-2.

Immunologic outcomes were available for 580 patients in the full data set and 469 in the strict time window data set (Supplementary Figure 1). For the primary outcome, 97.2% (95% CI, 95.9%–98.6%; 564/580) of study participants had an antibody response ≥100 U/mL, as assessed with the Elecsys S test and based on the full data set. This response was similar between Moderna mRNA-1273 (96.1% [95% CI, 93.7%–98.6%], 245/255) and Pfizer-BioNTech BNT162b2 (98.2% [95% CI, 96.7%–99.6%], 319/325; Table 2). These results were confirmed by the ABCORA 2, with an overall antibody response of 97.4% (95% CI, 96.1%–98.7%; 563/578) and immune response proportions comparable for mRNA-1273 (96.4% [95% CI, 94.2%–98.7%], 244/253) and BNT162b2 (98.2% [95% CI, 96.7%–99.6%], 319/325). When the ABCORA 2 sum S1 threshold of 17 was assessed, 95.0% (95% CI, 93.2%–96.8%; 549/578) had potentially neutralizing antibodies (mRNA-1273, 94.1% [95% CI, 91.2%–97.0%], 238/253; BNT162b2, 95.7% [95% CI, 93.5%–97.9%], 311/325). Similarly, all other immunologic outcomes revealed no difference between the vaccine products, and the analyses conducted on the strict time window data set (Supplementary Table 2) were in line with the findings of the full data set. At 8-week follow-up, 7.1% (95% CI, 5.0%–9.1%; 42/593) of participants reported that they tested positive for SARS-CoV-2. No severe COVID-19 episodes resulting in hospitalization or death occurred. Household members were SARS-CoV-2 positive in 4% of all cases (95% CI, 2.5%–5.6%; 24/593). Adverse events due to vaccine, such as systemic symptoms (eg, fever, headache) limiting normal daily activity, occurred in 10.2% (95% CI, 7.7%–12.6%; 60/589) of all participants (mRNA-1273, 13.4% [95% CI, 9.2%–17.5%], 35/262; BNT162b2, 7.6% [95% CI, 4.8%–10.5%], 25/327). Symptoms at injection sites limiting daily activities were reported by 7.0% of participants (95% CI, 4.9%–9.0%; 41/589). No deaths occurred, but 2 lung transplant recipients required hospitalization due to (1) worsening of general condition, fever, and dyspnea and (2) simultaneous viral pulmonary and gastrointestinal infection (both SARS-CoV-2 negative; Table 2). All outcomes stratified by cohort study (SHCS or STCS) are presented in Supplementary Table 3.

**Table 2.** Immunologic and Clinical Outcomes

	No. (%; 95% CI) or Mean (95% CI)			
Outcome	MRNA-1273 (Moderna)	BNT162b2 (Pfizer-BioNTech)	Total	Difference, %
Immunologic				
Antibody response				
Elecsys S, cutoff ≥100 U/mL	245/255 (96.1; 93.7–98.6)	319/325 (98.2; 96.7–99.6)	564/580 (97.2; 95.9–98.6)	−2.1 (−4.9 to 0.7)
Elecsys S, cutoff ≥0.8 U/mL	247/255 (96.9; 94.7–99.0)	321/325 (98.8; 97.6–100.0)	568/580 (97.9; 96.8–99.1)	−1.9 (−4.4 to 0.6)
ABCORA 2 [15]	244/253 (96.4; 94.2–98.7)	319/325 (98.2; 96.7–99.6)	563/578 (97.4; 96.1–98.7)	−1.7 (−4.4 to 1.0)
Neutralization prediction: ABCORA 2; cutoff sum S1 17 [15]	238/253 (94.1; 91.2–97.0)	311/325 (95.7; 93.5–97.9)	549/578 (95.0; 93.2–96.8)	−1.6 (−5.3 to 2.0)
IgG RBD	208.6 (199.1–218.1)	207.23(200.1–214.4)	207.8 (202.1–213.6)	
IgG S1	230.1 (218.1–242.0)	235.3 (225.6–244.9)	233.0 (225.5–240.5)	
IgA S1	5.4 (4.5–6.4)	5.4 (4.5–6.4)	5.4 (4.81–6.1)	
IgM S1	1.7 (1.2–2.2)	1.9 (1.4–2.4)	1.817 (1.5–2.2)	
Clinical^a^				
Confirmed SARS-CoV-2 infection	18/263 (6.8; 3.8–9.9)	24/330 (7.3; 4.5–10.1)	42/593 (7.1; 5.0–9.1)	
Asymptomatic	1/18 (5.6; 0–16.1)	3/24 (12.5; 0–25.7)	4/42 (9.5; .6–18.4)	
Symptomatic	17/18 (94.4; 83.9–100.0)	21/24 (87.5; 74.3–100.0)	38/42 (90.5; 81.6–99.4)	
Severe COVID-19^b^	0/263 (0.0)	0/330 (0.0)	0/593 (0.0)	
Confirmed SARS-COV-2 infection of household members	10/263 (3.8; 1.5–6.1)	14/330 (4.2; 2.1–6.4)	24/593 (4.0; 2.5–5.6)	
Safety^c^				
Hospitalization without SARS-CoV-2 infection	2/263 (0.8; 0–1.8)	0/330 (0.0)	2/593 (0.3; 0–.8)	
Death	0/263 (0.0)	0/330 (0.0)	0/593 (0.0)	
Any symptoms at injection site limiting daily activity 7 d following third vaccination	25/262 (9.5; 6.0–13.1)	16/327 (4.9; 2.6–7.2)	41/589 (7.0; 4.9–9.0)	
Any systemic symptoms limiting daily activities 7 d following third vaccination	35/262 (13.4; 9.2–17.5)	25/327 (7.6; 4.8–10.5)	60/589 (10.2; 7.7–12.6)	
Any vaccine-related symptoms leading to consultation 7 d following third vaccination	2/262 (0.8; 0–1.8)	0/327 (0.0)	2/589 (0.3; 0–.9)	

Abbreviations: ABCORA: Antibody Coronavirus Assay 2; Ig, immunoglobulin; RBD, receptor-binding protein.

^a^Clinical outcomes: 8 missing.

^b^Safety outcomes: 12 missing.

^c^Symptoms leading to hospitalization.

**Figure 1.**
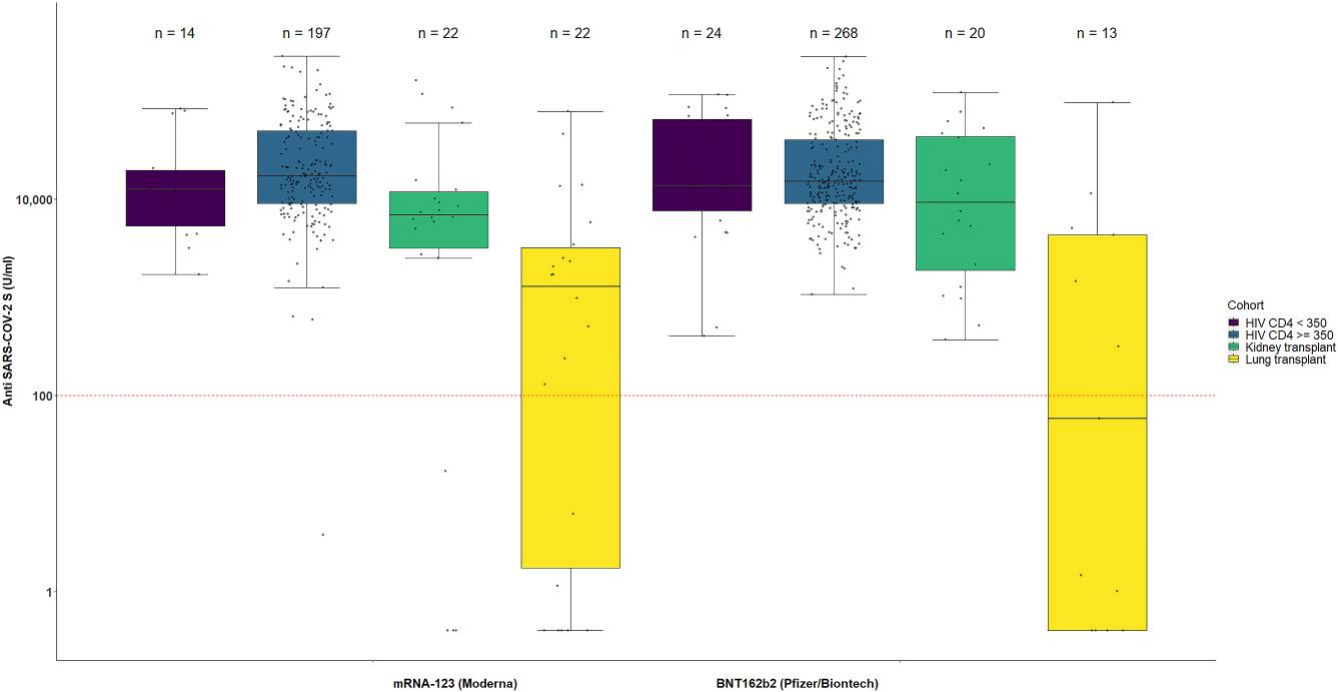
Antibody response in patients with immunocompromise after receiving the third SARS-CoV-2 vaccine via the Elecsys S test from Roche. SARS-CoV-2 spike protein receptor-binding domain antibody levels in patients who received the third SARS-CoV-2 vaccine and provided a blood sample at follow-up: people living with HIV with CD4 cell counts <350 or >350 cells/μL and recipients of kidney or lung solid organ transplantation. A value corresponding to half the detection limit (0.2 U/mL) is assigned to measurements below the detection limit (<0.4 U/mL). Horizontal black line, median; box, lower and upper quartiles; whiskers, all samples lying within 1.5 times the IQR.

We observed that all PLWH with the exception of 1, irrespective of CD4 cell count, had an antibody response ≥100 U/mL (Figure 1). The antibody response was lower in SOT recipients, especially in lung transplant recipients. Among all SOT recipients, 62 (80.5%; 95% CI, 71.7%–89.3%) showed an immune response ≥100 U/mL (Supplementary Table 3). Results from prespecified subgroup analyses (Supplementary Table 4) suggest that patients with lung transplants had a lower immune response (65.7% [95% CI, 50.0%–81.4%], 23/35) than kidney transplant recipients (92.9% [95% CI, 85.1%–100%], 39/42). Antibody response stratified for patients who had a SARS-CoV-2 infection after the third vaccination is presented in Supplementary Table 5.

The baseline characteristics of SOT recipient responders (ie, those with an antibody response ≥100 U/mL) and nonresponders (antibody response <100 U/mL) are presented in Table 3 and Supplementary Table 6. Nonresponders were older, had more history of cardiovascular disease or metabolic syndrome, and were on a more intensive immunosuppressive therapy than responders (Table 3). The proportion of SOT recipients with an antibody response strongly increased after the third SARS-CoV-2 vaccine dose (80.5% [95% CI, 71.7%–89.3%], 62/77) as compared with that after the second vaccine (22.7% [95% CI, 12.6%–32.8%], 15/66; Table 4).

**Table 3.** Vaccine Responders and Nonresponders in Solid Organ Transplant Recipients After Receiving the Third Dose SARS-CoV-2 Vaccination

	No. (%) or Median (IQR)		
Characteristic	Nonresponder (n = 18)	Responder (n = 59)	All (N = 77)
Vaccine			
Moderna (mRNA-1273)	11 (61.1)	33 (55.9)	44 (57.1)
Pfizer-BioNTech (BNT162b2)	7 (38.9)	26 (44.1)	33 (42.9)
Sex			
Male	9 (50.0)	33 (55.9)	42 (54.5)
Female	9 (50.0)	26 (44.1)	35 (45.5)
Age, y			
< 60	6 (33.3)	38 (64.4)	44 (57.1)
60–69	8 (44.4)	14 (23.7)	22 (28.6)
≥70	4 (22.2)	7 (11.9)	11 (14.3)
History of cardiovascular disease or metabolic syndrome			
No	3 (16.7)	15 (25.4)	18 (23.4)
Yes	15 (83.3)	44 (74.6)	59 (76.6)
Immunotherapy			
Dual therapy	1 (5.6)	7 (11.9)	8 (10.4)
Intense therapy	17 (94.4)	52 (88.1)	69 (89.6)
Glucocorticoids	15 (83.3)	50 (84.7)	65 (84.4)
Mycophenolate mofetil	16 (88.9)	47 (79.7)	63 (81.8)
Azathioprine	1 (5.6)	5 (8.5)	6 (7.8)
Cyclosporine	3 (16.7)	3 (5.1)	6 (7.8)
Tacrolimus	14 (77.8)	54 (91.5)	68 (88.3)
Time since transplant, d	1768 (762–2822)	1433 (234−2758)	1504 (343–2835)

Responder according to the full data set (Roche Elecsys Anti SARS-Cov2 S; primary outcome). Abbreviation: IQR: Interquartile range.

**Table 4.** Antibody Response After SARS-CoV-2 Vaccination: Second vs Third Dose

	No. (%; 95% CI)		
Antibody response	MRNA-1273 (Moderna)	BNT162b2 (Pfizer-BioNTech)	Total
After second vaccine dose^a^	178/204 (87.3; 82.7–91.8)	173/200 (86.5; 81.8–91.3)	351/404 (86.9; 83.6–90.2)
SHCS	169/170 (99.4; 98.3–100)	167/168 (99.4; 98.2–100)	336/338 (99.4; 98.6–100)
STCS	9/34 (26.5; 11.6–11.3)	6/32 (18.8; 5.2–32.3)	15/66 (22.7; 12.6–32.8)
After third vaccine dose^a^	245/255 (96.1; 93.7–98.6)	319/325 (98.2; 96.7–99.6)	564/580 (97.2; 95.9–98.6)
SHCS	210/211 (99.5; 98.6–100)	292/292 (100; 100–100)	502/503 (99.8; 99.4–100)
STCS	35/44 (79.6; 67.6–91.5)	27/33 (81.8; 68.7–95.9)	62/77 (80.5; 71.7–89.3)

Values were derived from the results of the first COVERALL trial (study 1).

Abbreviations: SHCS, Swiss HIV Cohort Study; STCT, Swiss Transplant Cohort Study.

^a^Elecsys S, cutoff ≥100 U/mL.

The order of vaccine products received is shown in Supplementary Table 7. The proportion of patients with an antibody response among participants who switched vaccines was comparable to those who received the same product for all 3 vaccines (Supplementary Table 8).

## DISCUSSION

Our results show that a high proportion of patients who were immunocompromised reached an antibody response ≥100 U/mL (Elecsys S test) after the third mRNA SARS-CoV-2 vaccine. Antibody response varied in patients with different levels of immunosuppression. Similar to results after the second SARS-CoV-2 vaccine [11], nearly all PLWH had an antibody response ≥100 U/mL irrespective of CD4 cell counts. Our results are in line with a recently published large systematic review and meta-analysis, concluding that the antibody response in PLWH is comparable to that of the general population [20]. For SOT recipients, our results showed that >80% of the included patients had an antibody response ≥100 U/mL after the third SARS-CoV-2 vaccine. This is in sharp contrast to the antibody response of SOT recipients after the second vaccine, where only 23% had an antibody response per the prespecified cutoff (≥100 U/mL) [11]. These findings support several other studies assessing the antibody responses in SOT recipients, confirming that these patients particularly benefit from the third SARS-CoV-2 vaccination [6, 14, 21–23]. For SOT recipients who still did not have a sufficient antibody response, it is unclear if additional vaccines may further increase the antibody response or if alternative strategies, such as prophylactic antispike monoclonal antibodies, should be prioritized if the circulating SARS-CoV-2 variants are susceptible [23–25]. Yet, the effectiveness of prophylactic monoclonal antibodies is under debate since the appearance of new SARS-CoV-2 omicron variants [26, 27].

In terms of safety, the third SARS-CoV-2 vaccine was well tolerated by patients from the SHCS and STCS. Systemic symptoms limiting daily activities were reported by 10% of patients, less than in our previous study assessing the second SARS-CoV-2 vaccine (16% with systemic symptoms [11]).

Our study has the following limitations. First, even though we recruited 601 participants, the sample size for SOT recipient nonresponders is too small to assess the factors associated with insufficient antibody response. As SOT recipients were prioritized when rollout [28] of the third SARS-CoV-2 vaccine began, we were not able to set up the study in time (ie, ensuring funding and ethical approval) and therefore missed a substantial number of eligible patients from the STCS. Adding a study center only partially compensated for this missed opportunity. Furthermore, the treatment of SOT recipients is strongly individualized, and the dosing scheme is not recorded in STCS and could not be included in our analysis. Second, while the majority of patients had a very good antibody response, we observed a considerable number of SARS-CoV-2 infections (42/593, 7.1%). This might raise some concerns about the relevance of our primary endpoint (ie, thresholds for antibody response are under debate [29, 30]) and the effectiveness of the vaccines in respect to new variants such as Omicron [31, 32]. Nevertheless, the vaccines did protect against severe COVID-19 [33–35]. This is confirmed by our study, where we did not observe any severe COVID-19 cases. Third, a large proportion of baseline blood samples were missing. Therefore, we could not compare the antibody response before and after the third vaccine. We also have limited knowledge about natural infections, which could have caused a rise in antibody response. Last, we did not assess T-cell response, and we were able to include only lung and kidney transplant recipients due to logistical reasons.

In conclusion, a high proportion of patients who were immunocompromised had an antibody response after the third SARS-CoV-2 vaccination, and relatively few vaccine-related adverse events were reported. SOT recipients profited substantially in terms of an increased antibody response between the second and third doses. For patients with low humoral response, alternatives have to be explored. The new bivalent SARS-CoV-2 mRNA vaccines may represent a promising approach.

## Supplementary Material

ofad536_Supplementary_Data

## Supplementary Data

Supplementary materials are available at *Open Forum Infectious Diseases* online. Consisting of data provided by the authors to benefit the reader, the posted materials are not copyedited and are the sole responsibility of the authors, so questions or comments should be addressed to the corresponding author.

## Notes

***Acknowledgment.*** We thank all cohort patients who voluntarily participated in this study. We are grateful for all work conducted at the local centers in Basel (Erol Cetinkaya, Patricia Francois, Diana Grauwiler, Tobias Kunz, Maria Pascarella, Rebekka Platter, Louise Seiler), Zürich (Daniela Gsell, Nino Scherrer, Herbert Kuster, Laura Tschuor, Rosita Nujic, Christina Grube, Flurina Brunschweiler, Rheliana Katzensteiner, Christine Schneider, Claudia Schmidt, Dominik Damm, René Hage), Bern (Daniela Hirter, Manuela Correia da Silva Saúde, Pia Scherler, Anna Rehbock, Cornelia Staehelin), Lausanne (Dela Golshayan, Nicolas Guggisberg, Ana Cipriano, Silvia Ciganek, Isabelle Regamey), and the Institute of Medical Virology, University of Zürich. Furthermore, we thank the ethical committee Nordwest- and Zentralschweiz, Switzerland, for handling this second COVERALL substudy in a very timely manner.

***Author contributions.*** B. S., H. C. B., F. C., I. A. A., H. F. G., A. R., M. T. K., M. B., and K. K. designed the study. K. K. was responsible for managing the data platform. F. C. conducted the statistical analyses and was responsible for monitoring. BS, A. G., C. M. S., A. T. H., A. Amstutz, and M. B. coordinated the study. P. A., M. P. S., A. L. E., B. H., D. L. B., M. M. S., T. F. M., M. T., A. R., H. F. G., M. P.-O., O. M., A. C., and N. J. M. were responsible for patient recruitment and follow-up at local centers. I. A. A., A. T., S. E., and A. Audigé conducted all laboratory analyses. A. G., B. S., A. Amstutz, F. C., and M. B. interpreted the data. A. G. and B. S. wrote the first draft of the manuscript. All authors read and approved the final version of the manuscript.

***Members of the SHCS.*** I. Abela, K. Aebi-Popp, A. Anagnostopoulos, M. Battegay, E. Bernasconi, D. L. Braun, H. C. Bucher, A. Calmy, M. Cavassini, A. Ciuffi, G. Dollenmaier, M. Egger, L. Elzi, J. Fehr, J. Fellay, H. Furrer, C. A. Fux, H. F. Günthard (president of the SHCS), A. Hachfeld, D. Haerry (deputy of Positive Council), B. Hasse, H. H. Hirsch, M. Hoffmann, I. Hösli, M. Huber, D. Jackson-Perry (patient representatives), C. R. Kahlert (chair of the Mother & Child Substudy), L. Kaiser, O. Keiser, T. Klimkait, R. D. Kouyos, H. Kovari, K. Kusejko (head of data center)., N. Labhardt, K. Leuzinger, B. Martinez de Tejada, C. Marzolini, K. J. Metzner, N. Müller, J. Nemeth, D. Nicca, J. Notter, P. Paioni, G. Pantaleo, M. Perreau, A. Rauch (chair of the Scientific Board), L. Salazar-Vizcaya, P. Schmid, R. Speck, M. Stöckle (chair of the Clinical and Laboratory Committee)., P. Tarr, A. Trkola, G. Wandeler, M. Weisser, S. Yerly.

***Members of the STCS.*** Patrizia Amico, John-David Aubert, Vanessa Banz, Sonja Beckmann, Guido Beldi, Christoph Berger, Ekaterine Berishvili, Annalisa Berzigotti, Isabelle Binet, Pierre-Yves Bochud, Sanda Branca, Heiner Bucher, Anne Cairoli, Emmanuelle Catana, Yves Chalandon, Sabina De Geest, Olivier De Rougemont, Sophie De Seigneux, Michael Dickenmann, Joëlle Lynn Dreifuss, Michel Duchosal, Thomas Fehr, Sylvie Ferrari-Lacraz, Christian Garzoni, Déla Golshayan, Nicolas Goossens, Fadi Haidar, Jörg Halter, Dominik Heim, Christoph Hess, Sven Hillinger, Hans H. Hirsch, Patricia Hirt, Linard Hoessly, Günther Hofbauer, Uyen Huynh-Do, Franz Immer, Michael Koller, Bettina Laesser, Frédéric Lamoth, Roger Lehmann, Alexander Leichtle, Oriol Manuel, Hans-Peter Marti, Michele Martinelli, Valérie McLin, Katell Mellac, Aurélia Merçay, Karin Mettler, Nicolas J. Mueller, Ulrike Müller-Arndt, Beat Müllhaupt, Mirjam Nägeli, Graziano Oldani, Manuel Pascual, Jakob Passweg, Rosemarie Pazeller, Klara Posfay-Barbe, Juliane Rick, Anne Rosselet, Simona Rossi, Silvia Rothlin, Frank Ruschitzka, Thomas Schachtner, Stefan Schaub, Alexandra Scherrer, Aurelia Schnyder, Macé Schuurmans, Simon Schwab, Thierry Sengstag, Federico Simonetta, Susanne Stampf, Jürg Steiger, Guido Stirnimann, Ueli Stürzinger, Christian Van Delden, Jean-Pierre Venetz, Jean Villard, Julien Vionnet, Madeleine Wick, Markus Wilhelm, Patrick Yerly.

***Patient consent statement.*** Each patient's written consent was obtained. The trial was approved by all local ethical committees and conforms to international standards. All protocols were approved by the ethical committee Nordwest- and Zentralschweiz, Switzerland (BASEC 2021-000593), and the full protocols are publicly available on trial registry (https://clinicaltrials.gov/ct2/show/NCT04805125).

***Disclaimer.*** The study design was discussed with the funder (Moderna) before funding was granted. The funders had no role in data collection and analysis, decision to publish, or preparation of the manuscript. Before submission, Moderna had the right to read the manuscript and suggest revisions; however, no revisions were suggested.

***Financial support*.** This work was supported by Moderna (COVERALL-2); the Swiss National Science Foundation (grant 31CA30_196245 for the setup of the trial platform and the first substudy [COVERALL]; grants 177499 and 201369 for Swiss HIV Cohort Study; grant 33CS30_177522 for STCS); the Pandemiefonds of the UZH foundation (a grant for the diagnostic work to A. T.); the Promedica Foundation (research grant 14851 M to I. A. A.); the Research Foundation of the University of Basel (grant to B. S.); the Janggen Pöhn Foundation and the Swiss National Science Foundation (personal grant 323530_221860 to C. M. S.).
