# Supplementary material for: Antibody Response After the Third SARS-CoV-2 Vaccine in Solid Organ Transplant Recipients and People Living With HIV (COVERALL-2)
Source: Open Forum Infect Dis. 2023 Nov 3;10(11):ofad536. doi: 10.1093/ofid/ofad536 (PMC10655940; doi:10.1093/ofid/ofad536)
Supplement: ofad536_Supplementary_Data [file ofad536_supplementary_data.docx]

**Appendix**

**Antibody response after the third SARS-CoV-2 vaccine in solid organ transplant recipients and people living with HIV (COVERALL-2)**

Alexandra Griessbach, Frédérique Chammartin, Irene A. Abela, Patrizia Amico, Marcel P. Stoeckle, Anna L. Eichenberger, Barbara Hasse, Dominique L. Braun, Macé M. Schuurmans, Thomas Müller, Michael Tamm, Annette Audigé, Nicolas J. Mueller, Andri Rauch, Huldrych F. Günthard, Michael T. Koller, Alexandra Trkola, Selina Epp, Alain Amstutz, Christof M. Schönenberger, Ala Taji Heravi, Matthaios Papadimitriou-Olivgeris, Alessio Casutt, Oriol Manuel, Katharina Kusejko, Heiner C. Bucher^,^, Matthias Briel, Benjamin Speich, and the Swiss HIV Cohort Study and the Swiss Transplant Cohort Study

1. **Eligibility Criteria**

Inclusion criteria:

- All patients with either a chronic HIV infection or recipients of solid organs registered with informed consent from the SHCS and STCS cohorts aged ≥18 years
- Patients with solid organ transplantation of lungs or kidneys at least one month post-transplantation with a prednisone dose of 20mg or less.
- Additional consent for participation in trial extension
- Third covid-19 vaccination recommended by treating physician and administered in the frame of clinical routine

Exclusion criteria:

- Pregnancy
- Acute symptomatic SARS-CoV-2 infection, influenza, or other acute respiratory tract infection
- Known allergy or contra-indications for vaccines or any vaccine components
- Any emergency condition requiring immediate hospitalization for any condition
- Patients with solid organ transplantation (lung or kidney) with the following conditions:
- Solid organ transplant recipients less than one month post-transplantation

- Solid organ transplant recipients with the use of T-cell depleting agents in the last 3 months (i. e induction treatment in standard risk or high-risk immunological situation or rejection treatment).

- Solid organ transplant recipients with the need of pulse corticosteroids (>100mg prednisone or

- equivalent) in the last 1 month or rituximab in the last 6 months

- Solid organ transplant recipients with the need of any kind of chemotherapy treatment for cancer

1. **Outcomes**

Immunological endpoints

*Primary endpoint:*

- The proportion of patients with a positive antibody (pan-Ig) response to SARS-CoV-2 spike (S1) protein receptor binding domain (RBD) in human serum or plasma assessed by the commercial immunoassay Elecsys Anti-SARS-CoV-2 S (Elecsys S) from Roche Diagnostics (1). An antibody response will be considered as positive using the threshold of ≥ 100 units/ml, predicting a protective immune response as indicated Khoury et al.[1] *Secondary endpoints:*
- The proportion of patients with a positive antibody response to SARS-CoV-2 RBD using the Elecsys S assay by Roche, using a threshold of ≥0.8 units/ml as defined by the manufacturer.
- The proportion of patients with a positive antibody response using the Antibody CORonavirus Assay (ABCORA) 2 that assesses seropositivity by measuring IgG, IgA and IgM responses to SARS-CoV-2 RBD, S1, S2 and N.
- The proportion of patients with neutralizing neutralization activity against the vaccine strain Wuhan-Hu-1 in sera, defined as having an ABCORA sum S1 (sum of S1 signal over cut-off values of IgG, IgA, IgM) above the threshold of 17.
- Mean IgG response to SARS-CoV-2 RBD, using ABCORA 2.
- Mean IgM, IgA and IgG responses to SARS-CoV-2 S1 using ABCORA 2.
- Newly PCR-confirmed asymptomatic SARS-CoV-2 infection. Clinical outcomes
- Newly PCR- or antigen confirmed SARS-CoV-2 infection
- -Newly PCR- or antigen confirmed asymptomatic SARS-CoV-2 infection
- Newly PCR- or antigen confirmed symptomatic SARS-CoV-2 infection
- -Severe COVID-19 infection, hospitalization due to COVID-19 or death
- Patient reported asymptomatic or symptomatic infections of household members.
- - any local symptom (redness or swelling or prolonged pain at injection side) limiting continuation of normal daily activities during the first 7 days after vaccination
- any systemic symptom (fever, generalized muscle or joint pain) limiting continuation of normal daily activities during the first 7 days after vaccination
- any vaccine related symptom leading to contacting a physician during the first 7 days after vaccination

Due to a shift in COVID-19 testing practices in Switzerland, the pre-specified clinical outcomes, “Newly PCR-confirmed SARS-CoV-2 infection” (outcomes a, b, c), were adapted to include SARS-CoV-2 infections confirmed by antigen tests

**Definition of metabolic syndrome:**

Metabolic syndrome is defined in SHCS as having any three of the following conditions: abdominal obesity (waist circumference>102 cm in men, >88 cm in women), triglyderides≥1.69 mmol/L, low HDL cholesterol (<1.03 mmol/L in men, <1.29 mmol/L in women), blood pressure ≥130/≥85 mmHg or diabetes) and in STCS as having any three of the following conditions hyperlipidemia, obesity, hypertension and diabetes.

**Figure S1 Flow Chart**

**
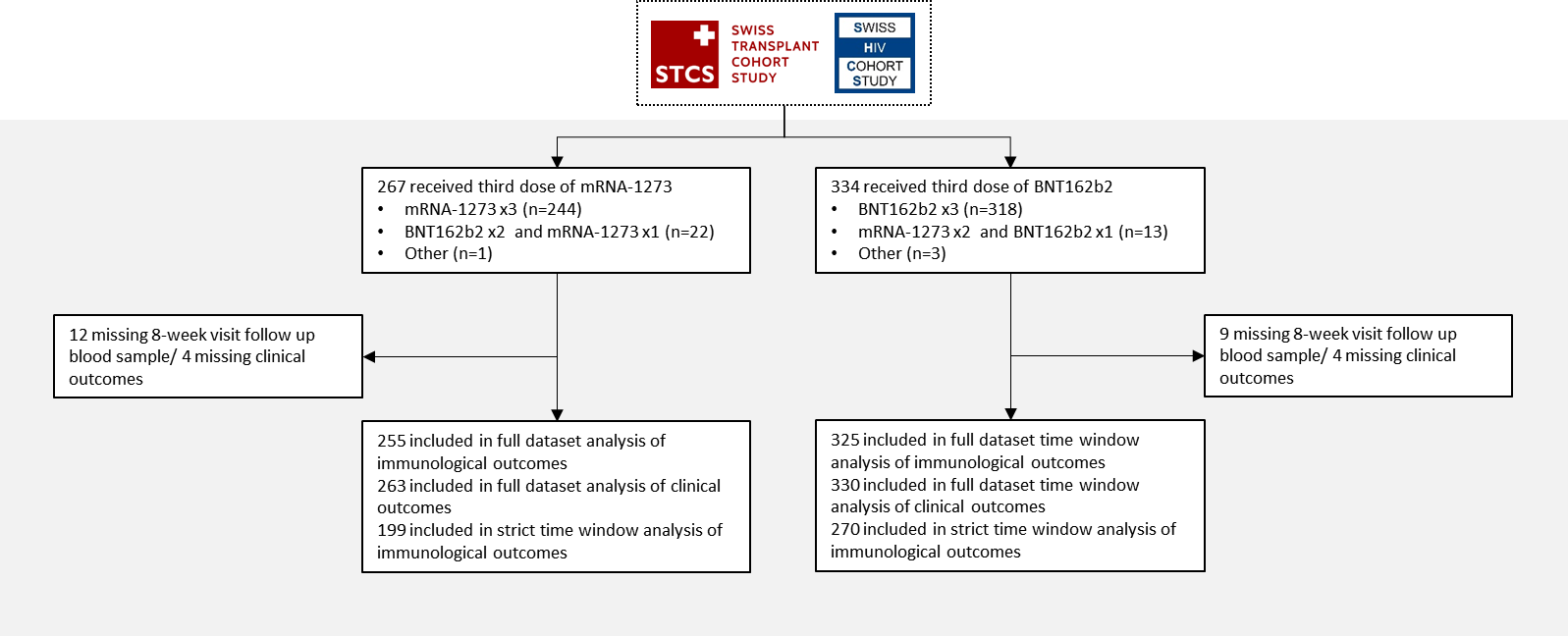
**

**Table S1:** Baseline characteristics stratified by participating cohort study

| **Characteristics** | **SHCS** | | | **STCS** | | |
| --- | --- | --- | --- | --- | --- | --- |
|  | **Moderna  (N=219)** | **Pfizer/Biontech (N=297)** | **Total  (N=516)** | **Moderna  (N=48)** | **Pfizer/Biontech  (N=37)** | **Total  (N=85)** |
| **Median age** | 54 (46-60) | 57 (47-64) | 56 (46-62) | 56 (39-65) | 60 (42-65) | 57 (40-65) |
| **Sex** |  |  |  |  |  |  |
| Male | 161 (73.5%) | 248 (83.5%) | 409 (79.3%) | 29 (60.4%) | 18 (48.6%) | 47 (55.3%) |
| Female | 58 (26.5%) | 49 (16.5%) | 107 (20.7%) | 19 (39.6%) | 19 (51.4%) | 38 (44.7%) |
| **History of cardiovascular disease or metabolic syndrome** |  |  |  |  |  |  |
| No | 155 (70.8%) | 198 (66.7%) | 353 (68.4%) | 10 (20.8%) | 9 (24.3%) | 19 (22.4%) |
| Yes | 64 (29.2%) | 99 (33.3%) | 163 (31.6%) | 38 (79.2%) | 28 (75.7%) | 66 (77.6%) |
| **CD4 cell count (cells/µL)^a^** |  |  |  |  |  |  |
| <350 | 15 (6.9%) | 25 (8.4%) | 40 (7.7%) | - | - | - |
| >350 | 204 (93.1%) | 272 (91.6%) | 476 (92.3%) | - | - | - |
| **Unsuppressed HIV viral load^ab^** |  |  |  |  |  |  |
| No | 212 (96.8%) | 288 (97.0%) | 500 (96.9%) | - | - | - |
| Yes | 7 (3.2%) | 9 (3.0%) | 16 (3.1%) | - | - | - |
| **Transplanted organc^c^** |  |  |  |  |  |  |
| Kidney transplant | - | - | - | 24 (50.0%) | 21 (56.8%) | 45 (52.9%) |
| Lung transplant | - | - | - | 24 (50.0%) | 16 (43.2%) | 40 (47.1%) |
| **Immunosuppressive therapy^c^** |  |  |  |  |  |  |
| Less intense (<2 regimen)^d^ | - | - | - | 6 (12.5%) | 5 (13.5%) | 11 (12.9%) |
| Intense (3 or 4 regimen)^d^ | - | - | - | 42 (87.5%) | 32 (86.5%) | 74 (87.1%) |
| **Nucleocapside protein^e^** |  |  |  |  |  |  |
| Non-reactive | 162 (87.1%) | 239 (85.7%) | 401 (86.2%) | 6 (100.0%) | 2 (33.3%) | 8 (66.7%) |
| Reactive | 24 (12.9%) | 40 (14.3%) | 64 (13.8%) | 0 (0.0%) | 4 (66.7%) | 4 (33.3%) |
| Missing | 33 (15.0%) | 18 (6.1%) | 51 (9.9%) | 42 (85.7%) | 31 (83.8%) | 73 (85.9%) |
| ^a^ Only considering patients from the Swiss HIV Cohort Study. | | | | | | |
| ^b^ Unsuppressed HIV viral load defined as >50 copies/ml | | | | | | |
| ^c^ Only considering patients from the Swiss Transplant Cohort Study. | | | | | | |
| ^d^  intense treatment defined as triple or quadruple immunosuppressive regimen vs. less intense immunosuppressive therapy defined as dual immunosuppressive regimen | | | | | | |
| ^e^ Elecsys N test reactive to nucleocapsid protein indicates previous contact to SARS-CoV-2. | | | | | | |
| Abbreviations: IQR=Interquartile range; SHCS=Swiss HIV Cohort Study; STCT= Swiss Transplant Cohort Study | | | | | | |

**Table S2:** Primary and secondary outcomes in strict time window population

| **Outcomes** | **mRNA-1273** | **BNT162b2** | **Total** | **Difference** |
| --- | --- | --- | --- | --- |
| Antibody response (Elecsys® S, cutoff ≥100 units/ml) | 195/199  (98.0%; 96.0-99.9%) | 268/270  (99.3%; 98.2-100%) | 463/469  (98.7%; 97.7-99.7%) | -1.3 (-3.5; 0.9) |
| Antibody response (Elecsys® S, cutoff ≥0.8 units/ml) | 196/199  (98.5%; 96.8-100%) | 268/270  (99.3%; 98.2-100%) | 464/469  (98.9%; 98-99.9%) | -0.8 (-2.7; 1.2) |
| Antibody response (ABCORA 2) | 195/198  (98.5%; 96.8-100%) | 268/270  (99.3%; 98.2-100%) | 463/468  (98.9%; 98-99.9%) | -0.8 (-2.7; 1.2) |
| Neutralisation (ABCORA 2.0), cutoff 17 | 193/198  (97.5%; 95.3-99.7%) | 264/270  (97.8%; 96.0-99.5%) | 457/468  (97.6%; 96. -99.0%) | -0.3 (-3.1; 2.5) |
| IgG RBD, mean (95%CI) | 219.8 (210.4-229.1%) | 213.7 (206.7-220.7%) | 216.3 (210.6-221.9%) |  |
| IgG S1, mean (95%CI) | 244.7(232.3- 257.1%) | 245.6 (236.0-255.1%) | 245.2 (237.6-252.8%) |  |
| IgA S1, mean (95%CI) | 5.1 (4.3-6.0%) | 5.1 (4.2-6.0%) | 5.1 (4.58-5.7%) |  |
| IgM S1, mean (95%CI) | 1.8 (1.2-2.4%) | 2.0 (1.4-2.6%) | 1.9 (1.5- 2.3%) |  |
| **Clinical outcomes^b^** |  |  |  |  |
| Confirmed SARS-CoV-2 infection | 13/197 (6.6%;3.1-10.1%) | 19/270 (7.0%;4.0-10.1%) | 32/467 (6.9%;4.6-9.1%) |  |
| Asymptomatic SARS-CoV-2 infection | 1/13 (7.7%; 0.0-22.2%) | 3/19 (15.8%; 0.0-32.2%) | 4/32 (12.5%; 1.0-24.0%) |  |
| Symptomatic SARS-CoV-2 infection | 12/13 (92.3%; 77.8-100.0%) | 16/19 (84.2%; 67.8-100.0%) | 28/32 (87.5%; 76.0-99.0%) |  |
| Severe COVID-19 infection^a^ | 0/197 (0.0%) | 0/270 (0.0%) | 0/467 (0.0%) |  |
| Confirmed SARS-COV-2 infection of household members | 9/197 (4.6%; 1.7-7.5%) | 13/27 (4.8%; 2.3-7.4%) | 22/467 (4.7%; 2.8-6.6%) |  |
| Hospitalizations | 0/197 (0.0%) | 0/270 (0.0%) | 0/467 (0.0%) |  |
| Death | 0/197 (0.0%) | 0/270 (0.0%) | 0/467 (0.0%) |  |
| **Safety outcomes^c^** |  |  |  |  |
| Any symptoms at injection site 7 days following 3rd vaccination | 16/196-(8.2%; 4.3-12.0% ) | 9/269 (3.3%; 1.2-5.5% ) | 25/465 (5.4%; 3.3-7.4%) |  |
| Any systemic symptoms limiting daily activities 7 days following 3rd vaccination | 26/196 (13.3%; 8.5-18.0%) | 17/269 (6.3%; 3.4-9.2%) | 43/465 (9.2%; 6.6-11.9%) |  |
| Any vaccine-related symptoms leading to consultation 7 days following 3rd vaccination | 2/196 (1.0%; 0.0-2.4%) | 0/269 (0.0%) | 2/465 (0.4%, 0.0-1.0%) |  |

^a^ Symptoms leading to hospitalisation

^b^clinical outcomes: missing for 3

^c^safety outcomes**:** missing for 5

Abbreviations: CI: Confidence Interval, Ig: Immunoglobulin

**Table S3:** Primary and secondary outcomes stratified by SHCS and STCS cohort

**Table S4 - Primary outcomes for solid transplant recipient stratification by transplant type and immunosuppressive therapy**

| **Outcomes** | **mRNA-1273** | **BNT162b2** | **Total** |
| --- | --- | --- | --- |
| Antibody response (Elecsys® S, cutoff ≥100 units/ml) lung transplant | 16/22  (72.7%; 54.1-91.3%) | 7/13  (58.9%; 26.8-81.0%) | 23/35  (65.7%; 50.0-81.4%) |
| Antibody response (Elecsys® S, cutoff ≥100 units/ml) kidney transplant | 19/22  (86.4%; 72.0-100%) | 20/20  (100%; 100-100%) | 39/42  (92.9%; 85.1-100%) |
| Antibody response (Elecsys® S, cutoff ≥100 units/ml) intense immunosuppressive therapy | 30/39  (76.9%; 63.7-90.2%) | 25/30  (83.3%; 70.0-96.7%) | 55/69  (79.7%; 70.2-89.2%) |
| Antibody response (Elecsys® S, cutoff ≥100 units/ml) less intense immunosuppressive therapy (dual therapy) | 5/5  (100%; 100-100%) | 2/3  (66.7%; 13.3-100%) | 7/8  (87.5%; 64.6-100%) |

**Table S5: Antibody response for patients with and without confirmed SARS-Cov2 infection after the thirds SARS-CoV-2 vaccination**

|  | **Patients without confirmed SARS-Cov2 infection** | | | **Patients with confirmed SARS-Cov2 infection** | | |
| --- | --- | --- | --- | --- | --- | --- |
| **Outcomes** | **mRNA-1273** | **BNT162b2** | **Total** | **mRNA-1273** | **BNT162b2** | **Total** |
| **Immunological outcomes** |  |  |  |  |  |  |
| Antibody response (Elecsys® S, cutoff ≥100 units/ml) | 225/235  (95.7%; 93.2-98.3%) | 293/299  (98.0%; 96.4-99.6%) | 518/534  (97.0%; 95.6-98.5%) | 18/18  (100.0%; 100.0-100.0%) | 24/24  (100.0%; 100.0-100.0%) | 42/42  (100.0%; 100.0-100.0%) |
| Antibody response (Elecsys® S, cutoff ≥0.8 units/ml) | 227/235  (96.6%; 94.3-99.9%) | 295/299  (98.7%; 97.4-100.0%) | 522/534  (97.8%; 96.5-99.0%) | 18/18  .0%; 100.0-10(1000.0%) | 24/24  (100.0%; 100.0-100.0%) | 42/42  (100.0%; 100.0-100.0%) |
| Antibody response (ABCORA 2) | 224/233  (96.1%; 93.7-98.6%) | 293/299  (98.0%; 96.4-99.6%) | 517/532  (97.2%; 95.8-98.6%) | 18/18  (100.0%; 100.0-100.0%) | 24/24  (100.0%; 100.0-100.0%) | 42/42  (100.0%; 100.0-100.0%) |
| Neutralisation prediction (ABCORA 2; cutoff sum S1 17) | 220/233  (94.4%; 91.5-97.4%) | 285/299  (95.3%; 92.9-97.7%) | 505/532  (94.9%; 93.1-96.8%) | 16/18  88.9% (74.4-100.0) | 24/24  (100.0%; 100.0-100.0%) | 40/42  (95.2%; 88.8-100.0) |
| IgG RBD, mean (95%CI) | 208.1 (198.2, 217.9) | 204.4 (196.8- 212.0) | 206.0 (199.9- 212.0) | 210.3 (166.5- 254.0) | 245.1 (232.5, 257.7) | 230.2 (210.4- 249.9) |
| IgG S1, mean (95%CI) | 227.6 (215.5- 240.0) | 230.5 (220.4-240.6) | 229.3 (221.5- 237.1) | 255.9 (197.5- 314.3) | 294.5 (270.7, 318.3) | 278.0 (250.3- 305.6) |
| IgA S1, mean (95%CI) | 5.2 (4.2- 6.2) | 5.4 (4.4- 6.4) | 5.3 (4.6- 6.0) | 8.5 (4.1- 12.8) | 6.3 (4.1- 8.4) | 7.2 (5.1- 9.4) |
| IgM S1, mean (95%CI) | 1.7 (1.2- 2.2) | 1.9 (1.3- 2.4) | 1.8 (1.4- 2.2) | 2.2 (0.0- 4.9) | 1.8 (0.7- 2.8) | 2.0 (0.7- 3.2) |

**Table S6:** **Baseline characteristics in responders and non-responders in solid organ transplant recipients**

**Table S7 Combination of vaccine products by cohort**

| **Product Combination** | **SHCS (N=516)** | **STCS (N=85)** | **Overall (N=601)** |
| --- | --- | --- | --- |
| **Moderna (3x)** | 206 (39.9%) | 38 (44.7%) | 244 (40.6%) |
| **Moderna (2x) followed by Pfizer-BioNTech (1x)** | 13 (2.5%) | 0 (0%) | 13 (2.2%) |
| **Moderna (1x) followed by Pfizer-BioNTech (2x)** | 1 (0.2%) | 0 (0%) | 1 (0.1%) |
| **Pfizer-BioNTech (3x)** | 282 (64.7%) | 36 (42.4%) | 318 (52.9%) |
| **Pfizer-BioNtech (2x) followed by Moderna (1x)** | 13 (2.5%) | 9 (10.6%) | 22 (3.7%) |
| **Missing information (2x) followed by Moderna (x1)** | 0 (0%) | 1 (1.2%) | 1 (0.1%) |
| **Missing information (2x) followed by Pfizer-BioNTech (1x)** | 0 (2.5%) | 1(1.2%) | 1 (0.1%) |
| **Johnson Johnson (1x) followed by Pfizer-BioNTech (1x)** | 1 (0.2%) | 0 (0%) | 1 (0.1%) |

**Table S8 Antibody response for vaccine switchers**

| **Vaccine Combinations** | **SHCS Antibody response (%)** | **STCS _Antibody response (%)** | **Total Antibody response (%)** |
| --- | --- | --- | --- |
| **Moderna (3x)** | 197/198  (99.5%; 98.5-100.0%) | 28/36  (77.8%; 64.2-91.4%) | 225/234  (96.1%; 93.7-98.6%) |
| **Pfizer-BioNTech (3x)** | 276/276  (100.0%; 100.0-100.0%) | 26/33  (78.8%; 64.8-92.7%) | 302/309  (97.7%; 96.1-99.4%) |
| **Moderna (2x) followed by Pfizer-BioNTech (1x)** | 12/12  (100.0%; 100.0-100.0%) | 0/0  (0%; 0.0-0.0%) | 12/12  (100.0%; 100.0-100.0%) |
| **Pfizer-BioNtech (2x) followed by Moderna (1x)** | 12/12  (100.0%; 100.0-100.0%) | 5/8  (62.5.0%; 29.0-96.1%) | 17/20  (85.0%; 69.4-100.0%) |

**References**

1. Khoury DS, Cromer D, Reynaldi A, et al. Neutralizing antibody levels are highly predictive of immune protection from symptomatic SARS-CoV-2 infection. Nat Med. **2021**; 27(7):1205–1211.
